# Framing with facts: How frames shape evidence use in Switzerland’s pesticide policy discourse

**DOI:** 10.1007/s11077-026-09611-1

**Published:** 2026-05-21

**Authors:** Ueli Reber, Karin Ingold

**Affiliations:** 1 Eawag: Swiss Federal Institute of Aquatic Science and Technology, Dübendorf, Switzerland; 2 University of Bern, Bern, Switzerland; 3 Oeschger Center for Climate Change Research, University of Bern, Bern, Switzerland

**Keywords:** Framing, Evidence use, Policymaking, Pesticide policy, Political discourse, Content analysis, Knowledge utilisation, Switzerland

## Abstract

How evidence is used to frame policy issues plays a crucial role in shaping which knowledge is deemed relevant to policymaking. Drawing on argumentation theory and framing literature, we argue that evidence functions as a tool that speakers use strategically and systematically to support specific elements of frames (regarding causes, consequences, or solutions), thereby determining which bodies of knowledge are mobilised in political discourse. Using quantitative content analysis of media, trade, and parliamentary discourse in Switzerland from 2013 to 2022, we identified four frames relating to the issue of pesticide risk reduction: health, environment, economy, and practice. We found that across all frames, the most emphasised frame element is also the one most likely to be supported by evidence. Overall, consequence claims were more likely to be backed by evidence than claims about causes or policy solutions. These patterns reveal which bodies of knowledge are mobilised in political discourse in support of certain policy stances and where the presented frames lack supporting evidence. Recognising the role of framing in evidence use can thus support more transparent and reflexive policymaking by helping researchers and policymakers to identify unavailable or overlooked knowledge and addressing gaps in both research and policymaking.

## Introduction

At a substantive level, policymaking is a process of interpretation and persuasion in which the way an issue is framed shapes how it is understood, debated, and ultimately addressed (Koon et al., 2016; Rein & Schön, 1996). In this context, framing in itself is a process in which certain aspects of a perceived reality are selected and emphasised to increase their salience in order to promote a particular problem definition, identify causes, assess consequences, and propose solutions (Entman, 1993). The strategic framing of an issue by proponents and opponents of policy change therefore plays a critical role in influencing agenda setting, policy formulation, and decision-making processes (Baumgartner et al., 2009; Lakoff, 2010).

A critical aspect of the framing process is the use of evidence (Nisbet & Newman, 2015; Stucki & Sager, 2018)—factual knowledge that is presented to validate presented claims (Majone, 1989). As the subsequent sections will show, evidence lends legitimacy and authority to claims and consequently to a particular frame, thereby making them more persuasive (Boswell, 2009; Toulmin, 2003). Hence, evidence is often used strategically to frame a policy issue (Westberry, 2024; Yanovitzky & Weber, 2020). From a communication perspective, linking factual knowledge and normative statements to frame policy issues decisively influences knowledge’s role in and impact on policymaking, since it is only this linking that gives knowledge political relevance and meaning (Greenhalgh & Russell, 2009; Majone, 1989; Parkhurst, 2012).

To better understand how knowledge becomes politically relevant, we examined the use of evidence to frame a policy issue, specifically considering how different frames mobilise different bodies of knowledge. Although the connection between framing and evidence use in policymaking is certainly not a new discovery (Weiss, 1979) and has been highlighted previously (Cairney, 2019; Druckman & Lupia, 2017; Hansen, 2011; Nisbet, 2009), few empirical studies have been conducted to describe (Stucki & Sager, 2018; Yanovitzky & Weber, 2020) or investigate its mechanisms (Moerschel et al., 2023; Parkhurst, 2012; Wolf & Van Dooren, 2017). In this article, we aim to expand the literature by examining how evidence is used to frame pesticide risk reduction in Switzerland, thereby enhancing our understanding of the role frames play in embedding knowledge in policymaking.

Using quantitative content analysis, we collected data from three key arenas: mass media, agricultural trade media, and parliamentary debates. Pesticide risk reduction is a complex and contested issue that intersects with concerns over food production, environmental sustainability, and public health, often involving conflicting goals (Ingold et al., 2023). As a ‘wicked’ problem with no single definition or simple solution (Rittel & Webber, 1973), it provided an ideal case for studying framing and evidence use because different actors emphasise different aspects of the issue according to their priorities and interests. Switzerland is a particularly valuable case for this investigation. Its direct-democratic institutions, multilingual federal structure, and relatively autonomous parliament create conditions in which a wide range of actors engage publicly in the framing of a contested policy issue, often drawing on scientific or experiential evidence to justify their positions (Reber et al., 2025a). Moreover, the Swiss pesticide debate unfolded across multiple arenas and timescales—including a high-salience referendum campaign in 2021—allowing us to examine evidence use under varying levels of public attention. While the specific frames identified are case-specific, the theoretical argument and methodological approach developed here are transferable to other contested policy domains in which scientific knowledge and competing normative positions intersect.

Beyond improving our understanding of the role frames play in evidence use, this article contributes to the literature in at least two ways. First, by proposing and testing a theoretical argument on how evidence use relates to framing, we advance the understanding of the functions that evidence serve in policymaking (e.g. Boswell, 2009; Shaxson & Boaz, 2021), as well as the understanding of the barriers to its use (Capano & Malandrino, 2022; Farley-Ripple et al., 2020; Hofmann et al., 2023). In doing so, we contribute to both the framing literature (Walter & Ophir, 2024) and the literature on the politics of evidence-informed policymaking (Cairney, 2016). Second, by introducing and applying a novel data collection method for examining the use of evidence in framing, we contribute to the methodological toolbox of scholars interested in assessing policymakers’ use of evidence (e.g. Stucki & Sager, 2018; Yanovitzky & Weber, 2020). Based on content analysis of media articles and policy documents, we studied actual instances of evidence use, complementing research on knowledge uptake by political actors, which has typically been investigated through surveys or interviews (Ouimet et al., 2023). This enhances our understanding of how evidence is received and interpreted by policy actors and how they use it in political discourse, thus providing insight into an often overlooked aspect of evidence use in the policymaking process (Hofmann et al., 2023).

In the following sections, we first develop our theoretical argument about how diverse frames contribute to the relevance of different bodies of knowledge in policymaking. We then test it using data from the Swiss political discourse on pesticide risk reduction. To do so, we first explain our research design and how we collected data regarding frames and evidence used in framing. Finally, we present the findings and discuss their implications for both research and evidence-informed policymaking.

## Theory

The literature on framing is often described as ambiguous and diffuse (König, 2021), mainly because the term is used to describe and study different phenomena. In this context, Scheufele (1999) distinguished between media and individual frames, the former referring to structures in political discourse and the latter to mental structures in individuals’ minds. The two levels are interrelated in that the issue-related frames of political discourse influence how individuals perceive, organise, and interpret new information and the conclusions they draw from it (Pan & Kosicki, 1993; Scheufele, 1999). Discordance among frames thus forms the basis of policy contestation (Koon et al., 2016; Parkhurst, 2012). For this reason, political scientists have long been interested in studying media frames.

Among the diverse definitions of frames and framing, Entman’s (1993) definition has emerged as the most influential for studies concerned with the communication of frames—that is, the framing process (Walter & Ophir, 2024). Entman (1993) defined framing as the process of selecting and emphasising certain aspects of a perceived reality to promote a particular problem definition, causal interpretation, moral evaluation, and/or recommended solution. A frame thus comprises multiple elements: it identifies a problem, attributes a cause, makes moral judgements, and/or suggests solutions. For example, based on the empirical case we examined, policymakers might frame pesticide use as a public health concern due to its associated risks to call for stricter regulations regarding their authorisation and application.

Typically, framing does not mean conveying an entire frame all at once. Rather, combinations of the frame’s individual elements are communicated repeatedly over time and begin to form a recognisable pattern (Matthes & Kohring, 2008). For the health frame, this could mean, for example, that one statement problematises the health risks of pesticides, while another calls for stricter regulations to protect pesticide users and bystanders. Through repeated and varied exposure to such statements, individuals learn to understand them as elements of a larger pattern—a frame. Frames thus structure political discourse by determining whether and how a problem is defined and what solutions are considered (Dodge & Metze, 2024). Consequently, they constitute a policy issue and are thus integral to policymaking (Rein & Schön, 1996).

How does evidence come into play? To answer this question, we need to consider exactly how frame elements are communicated, for which argumentation theory can offer useful insights. Drawing from Toulmin (2003), the skeleton of an argument consists of three interlinked elements: the claim, the evidence (or grounds), and the warrant. The claim (C) is the assertion of a speaker intended to persuade others to accept it. The evidence (E) comprises the facts that provide a foundation for the claim. The warrant (W) is the underlying principle, rule, or assumption that explains why the data support the claim. Toulmin (2003, p. 92) illustrated their relationship as follows:

**Figure:**
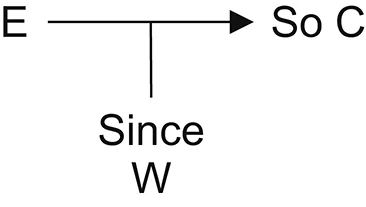

Here, frame elements take the form of claims (e.g. the claim that pesticides are a health hazard) comprising part of the health frame mentioned previously. While the warrant is rarely stated explicitly, speakers are likely to present relevant evidence to support their claims. That is, they use factual knowledge to prove that their claims are true or valid (Majone, 1989; Yanovitzky & Weber, 2020). For example, studies showing that exposure to pesticides increases the risk of cancer may be used as evidence to support the claim that pesticides are a health hazard.

**Figure:**
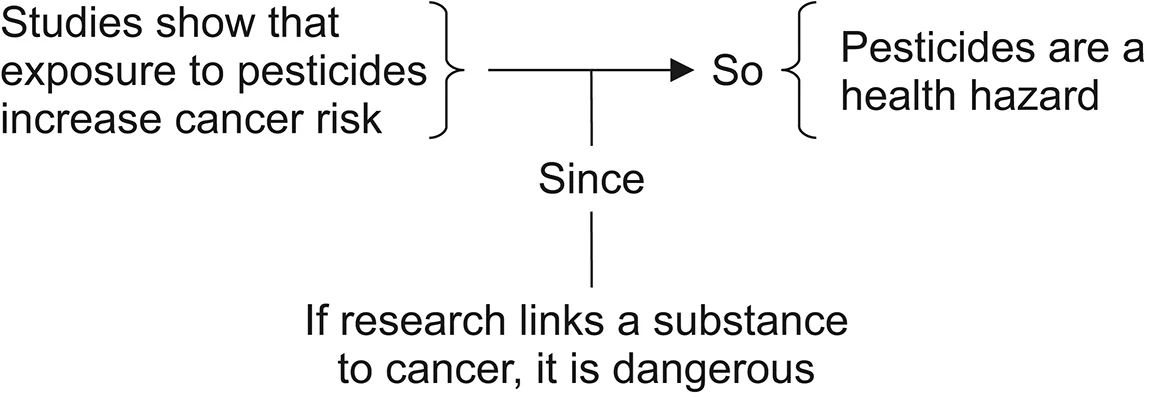

The use of evidence is, of course, influenced by many factors, such as the motivation of the speakers (Boswell, 2008; Hofmann et al., 2023), their access to knowledge, their capacity to process it (Newman et al., 2017), and the political and institutional context in which a statement is made (Cairney, 2016; Durrant et al., 2024; Mosley & Gibson, 2017; Reber et al., 2025a). However, functionally, evidence is always used to enhance the credibility and persuasiveness of a claim (Majone, 1989). This holds true even for claims that appear purely informative, as their underlying aim is still to persuade the audience of the accuracy of the stated facts—for example, that exposure to pesticides increases cancer risk. Moreover, because the use of evidence is inherently persuasive, it always contributes to a particular framing by selectively highlighting certain aspects of an issue and reinforcing a specific interpretation. This supports the notion that ‘there is no such thing as unframed information’ (Nisbet & Newman, 2015, p. 361) and that science communication is, in essence, always political communication (Scheufele, 2014).

For the framing process, this means that evidence is a tool that speakers can use to support their claims, thereby strengthening specific elements of the frame they wish to promote (Yanovitzky & Weber, 2020). However, different frames and their elements require distinct bodies of knowledge to serve as evidence. For example, framing an issue as a health concern requires that speakers draw on knowledge about health consequences, such as medical risks from pesticide exposure, whereas framing it as an economic or environmental concern requires evidence related to financial costs or environmental harm. Similarly, emphasising solutions rather than problems means using knowledge about effective policies and practices rather than problem-oriented knowledge about the causes and consequences of phenomenon (Reber et al., 2025b). Consequently, we argue that evidence is used both strategically and systematically to frame policy issues (Stucki & Sager, 2018; Wolf & Van Dooren, 2017), specifically to emphasise the key elements of frames and thereby attribute political relevance to particular bodies of knowledge.

**Fig. 1:**
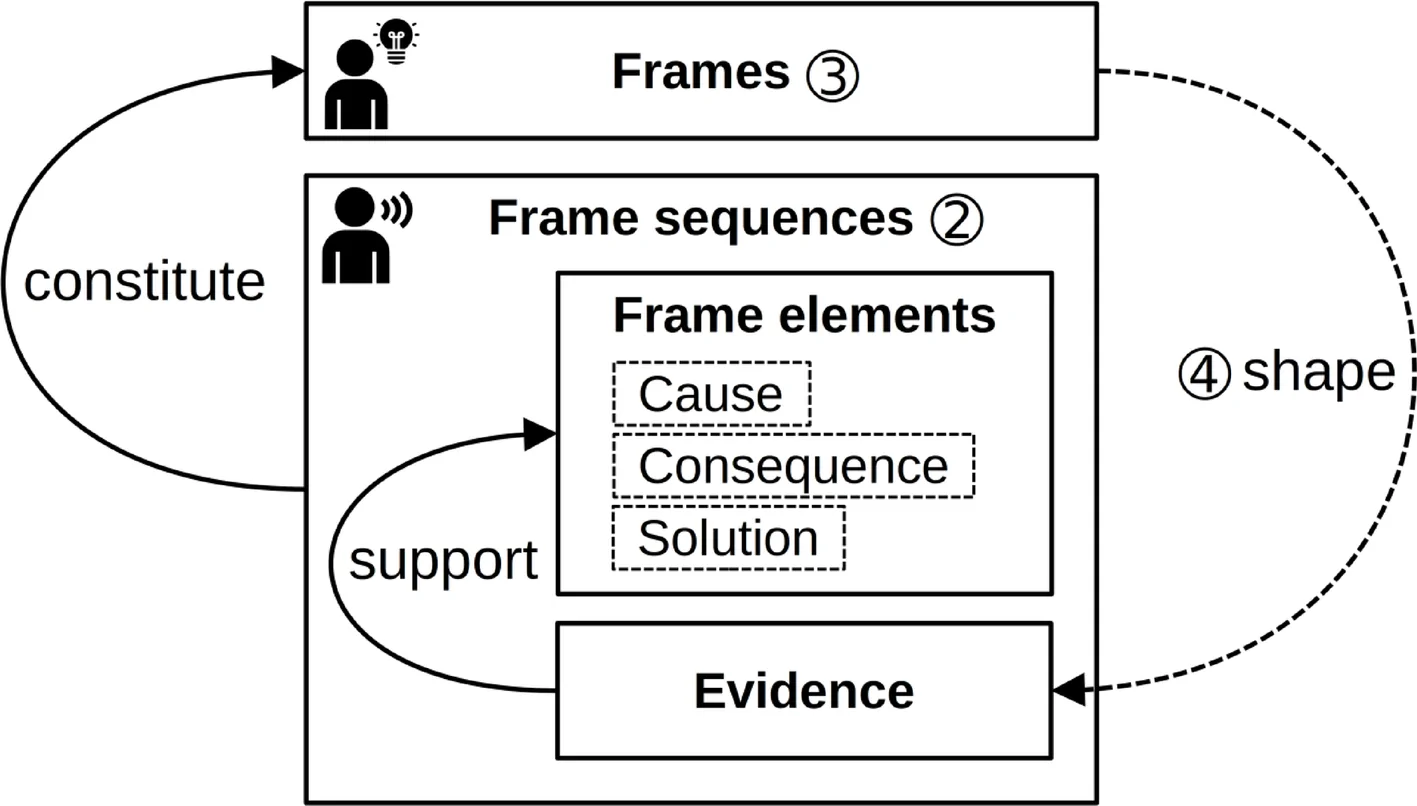
Overview of the concepts used to examine how evidence is used as a tool to frame the issue of pesticide risk reduction in Switzerland. Steps of the four-step research process are indicated. In a document (e.g. a mass media article), a speaker makes a statement (frame sequence) including one or multiple frame elements (cause, consequence, solution). Each frame element may or may not be supported by evidence. The sum of all frame sequences constitutes recurring patterns that individuals learn to recognise as frames. In this paper, we asses how these patterns (frames) shape how evidence is used in frame sequences

Clearly, the evidence used to frame a policy issue does not indicate which came first—the frame or the evidence—or whether a speaker adopted a particular frame based on available evidence or selected evidence to support an already established frame. However, examining the evidence used in framing an issue reveals what knowledge is considered most relevant for policymaking concerning that issue (Hawkins & Ettelt, 2019). Thus, it is not the knowledge itself that determines its relevance to policymaking, but the extent to which it resonates with the frames constituting a policy issue (Greenhalgh & Russell, 2009). To understand the relevance of knowledge to policymaking, it is thus necessary to understand how different ways of framing a policy issue mobilise specific bodies of knowledge as evidence. For this reason, we treat frames as the driving force behind the use of evidence in this paper, adopting the perspective that speakers seek to promote a particular frame by presenting supporting evidence where it serves this purpose (see also Fig. 1).

The policy framing literature has emphasised that frames shape not only how an issue is understood but also which policy responses are considered legitimate and relevant (Cairney, 2016; Koon et al., 2016). Drawing on the policy cycle as an analytical heuristic (Howlett & Ramesh, 1995), problem-oriented frames foreground causes and consequences and are most relevant when it comes to agenda-setting, while solution-oriented frames are more relevant at the policy formulation stage. Accordingly, different bodies of knowledge are likely to be mobilised: problem-oriented frames draw on knowledge about the causes and consequences of a policy issue, whereas solution-oriented frames require knowledge about the effectiveness and feasibility of policy options. The science communication literature similarly suggests that evidence use is strategic: speakers select evidence that maximises the persuasiveness of their most central claims (Nisbet, 2009; Scheufele, 2014). For these reasons, we expect evidence to be used primarily in support of the element that is foregrounded most strongly within a given frame.

## Methods

In this empirical study, we examined how evidence is used to frame the issue of pesticide risk reduction in Switzerland, focusing on how frames attribute relevance to knowledge about the causes, consequences and possible solutions of distinct problems. To this end, we employed a four-step process (Fig. 1): (1) sampling pesticide-related documents from three arenas (mass media, trade media, and parliament) covering the 10-year period 2013–2022; (2) quantitative content analysis of frame sequences and supporting evidence presented in the sampled documents;^1^ (3) identification of frames constituting the issue of pesticide risk reduction by clustering the frame elements coded in the previous step across all of the identified frame sequences (Matthes & Kohring, 2008); and (4) modelling the evidence used to support individual frame elements (i.e. causes, consequences, and solutions) based on the frame to which they contributed. In the following sections, we provide a more detailed discussion of each step of the process.

### Case selection and sampling (step 1)

Reducing pesticide risks is a sustainability challenge that requires policymakers to balance potentially conflicting health, environmental, and economic objectives (Möhring et al., 2020). Consequently, it is a contested policy issue that makes framing crucial for actors seeking to advance their own interests. Extensive scientific and experiential knowledge exists regarding both the benefits and risks of pesticide use: although pesticides can reduce crop losses and control vector-borne diseases, they also contribute to pesticide resistance in pests, harm nontarget organisms, contaminate ecosystems, and pose health risks to humans (EEA, 2023; Fuhrimann et al., 2022; UNEP, 2022). Farmers’ experiences and perceptions, particularly regarding economic impacts, health risks, and pest management strategies (Deguine et al., 2021), provide another important source of knowledge (Reber et al., 2025a). Nevertheless, uncertainties remain, especially concerning the long-term effects of pesticide mixtures and the outcomes of different policy interventions (Dueri & Mack, 2024; Nagy et al., 2020). The combination of abundant knowledge and persistent uncertainty, together with probably necessary trade-offs between competing objectives, made the political discourse on pesticide risk reduction an ideal case for studying how actors strategically and systematically used evidence to frame the issue.

Over the past 2 decades, Swiss pesticide policy has undergone a series of incremental changes (Barth, 2023), including the introduction of stricter criteria for the approval, sale, and use of pesticides, as well as a national action plan aimed at halving the risks of pesticide use, promoting alternatives, and protecting water resources. Simultaneously, Switzerland has also considered proposals for radical policy change, most notably through two popular initiatives calling for a general ban on synthetic pesticides and only giving subsidies to pesticide-free farms (Finger, 2021). Although both initiatives were rejected in the associated 2021 referenda, they triggered an intense, high-salience public debate (FÖG, 2021) and prompted political responses, including a more moderate parliamentary counterproposal. Policy change in Switzerland is often initiated by relatively radical proposals, followed by the formation of a more widely accepted compromise. This process requires political actors to engage in both problem- and solution-oriented discourse, as well as in different arenas. More specifically, Switzerland’s political system is characterised by direct democratic instruments and a comparatively autonomous parliament (Vatter, 2018). This requires actors across the political spectrum to engage publicly with competing frames, often drawing on scientific or experiential evidence to support their claims (Reber et al., 2025a).

This was reflected in the public debate on the pesticide issue in various arenas, including mass media (Kaiser, 2023), parliament (Reber et al., 2025b), and agricultural trade media (Zysset, 2023). Unlike mass media, which reflect broader public discourse (Ferree et al., 2002), and parliament, which records institutional deliberation (Bächtiger, 2014), trade media target sector-specific audiences, such as farmers and industry professionals, to reveal the perspectives of those most affected by pesticide policy. Therefore, we considered that analysing public communication on how and whether to reduce pesticide risk in Switzerland from 2013 to 2022—when the issue received varying levels of attention across three distinct arenas—would provide valuable insights into how evidence was used strategically in framing the policy issue.

Specifically, we compiled three corpora of documents mentioning pesticides that were published between 2013 and 2022.^2^ For mass media, we collected articles from five high-circulation, subscription-based Swiss newspapers published in German and French, using the Swissdox@LiRI media database.^3^ Articles from trade media were gathered from three widely read subscription-based publications in both languages, obtained directly from the publishers.^4^ Parliamentary documents, including acts and transcripts, were retrieved via the Swiss Parliament’s application programming interface. From these corpora, we then drew four samples: (1) a baseline mass media sample covering 2013–2022, excluding the 12 weeks before the June 2021 referenda on the two popular initiatives; (2) a high-salience mass media sample covering only those 12 weeks; (3) a 10-year trade media sample; and (4) a 10-year parliament sample. We calculated the sample sizes to achieve a 90% confidence level (see Table 1 in the appendix). To ensure balanced representation over time, we stratified the 10-year samples by year. The 12-week referendum campaign period was excluded from the baseline mass media sample because campaign coverage is likely to be both qualitatively and quantitively distinct from the ordinary media discourse. Treating this period as a separate sample rather than excluding it entirely allows us to compare evidence use under heightened and routine conditions.

### Content analysis of frame sequences (step 2)

To examine how evidence was used to frame the pesticide issue in Switzerland, we conducted a manual content analysis centred on what we referred to as *frame sequences*, defined as statements made by any speaker in the sampled documents that problematised the risks of pesticide use or pesticide use reduction. In addition to the problematisation aspect (moral judgement), a frame sequences could include claims about causes, consequences, or solutions—reflecting Entman’s (1993) definition of the elements of a frame. For example, a speaker might claim that pesticides endanger users due to acute poisoning (consequences) or argue that banning pesticides threatens food security (causes and consequences). A frame sequence could consist of one or more elements, which may not be part of a single, continuous statement. For example, in a media article, the same speaker might be quoted across several paragraphs, with each quote contributing to the speaker’s overall framing of the issue. Furthermore, a frame element could be conveyed through one or multiple claims (e.g. when a speaker highlighted different health risks associated with pesticide use). Our analysis focused on frame sequences in which speakers used evidence to support at least one of the frame elements presented.

Specifically, we followed the approach proposed by Matthes and Kohring (2008) to code the presence of predefined frame elements.^5^ In doing so, we distinguished between two problem definitions that a frame sequence could reflect: the risks of pesticide use and the risks of reducing pesticide use. Based on the problem definition, we coded the consequences (health, environmental, and economic), causes (political, economic, and practical), and solutions (policy, practical, and unspecified) presented in the frame sequences. Additionally, we recorded whether a speaker agreed or disagreed with a frame element (e.g. whether a specific health-related consequence of pesticide use was considered likely or unlikely). For each frame element, we recorded evidence as present if at least one associated claim was supported by evidence but as absent if none were.^6^ Finally, we collected data about the speakers in each frame sequence, specifically the actor type (e.g., political, economic, or civil society). An in-depth description of the coding process and all variables and their categories can be found in the codebook (Reber et al., 2023).^7^

### Identification of frames in discourse (step 3)

As a third step, we identified the frames constituting the issue of pesticide risk reduction by clustering their elements across all the frame sequences coded in the previous step. This approach, proposed by Matthes and Kohring (2008), rests on the idea that frames comprise recurring patterns of elements. Individuals recognise these patterns through repeated exposure over time, which allows them to identify particular frames, even when they are presented partially or incompletely. Specifically, we used hierarchical clustering to identify four frames, grouping together 588 frame elements drawn from 293 frame sequences. Initially, we constructed dummy variables for each frame element category. In line with Matthes and Kohring (2008), we excluded infrequent categories, such as the general/unspecific category of the cause and consequence variables, to reduce noise. We then applied the Ward method with Jaccard distance to cluster the reduced dataset. Based on the interpretability of the resulting clusters, we selected a four-cluster solution, identifying an economy, environment, health, and practice frame. Based on the clustering, we categorised each frame sequence as contributing to one of these frames.

### Modelling evidence use in framing (step 4)

To finally assess how evidence was used to frame the issue of pesticide risk reduction in Switzerland, we fitted a binomial regression model to predict evidence use for each frame element in our data based on the frame to which it contributed. That is, we used the frame assigned to each frame sequence in the previous step (economy, environment, health, and practice), along with the type of frame element (cause, consequence, and solution), as predictor variables to model whether a speaker used evidence to support it. We focused on consequences, causes, and solutions as frame elements because, based on our coding logic (Step 2), evidence cannot directly speak to the problem definition (i.e. the risks of pesticide use and reducing pesticide use), but it can support what is considered the cause, consequence, or solution. In addition, we controlled for several other factors that could potentially influence evidence use, including the speaker’s position regarding the frame element (support or no support), the number of frame elements mentioned by the speaker in the same frame sequence (1–3), the arena (mass media baseline, mass media high-salience, trade media, or parliament), the actor type of the speakers (civil society, economic, knowledge, media, political, or other), and the document language (French or German). Finally, we controlled for interactions between the frame element variables and each predictor variable, since evidence use may vary depending not only on the frame element and the frame it contributes to, but also on other factors, such as the speaker’s stance regarding it. To report the results, we used the model to calculate average marginal effects. This means that we used the model to make predictions for each observation in our dataset and then averaged these predictions across the predictor variables of interest (e.g. frame elements and frames).

## Results

### Frames in discourse

**Fig. 2:**
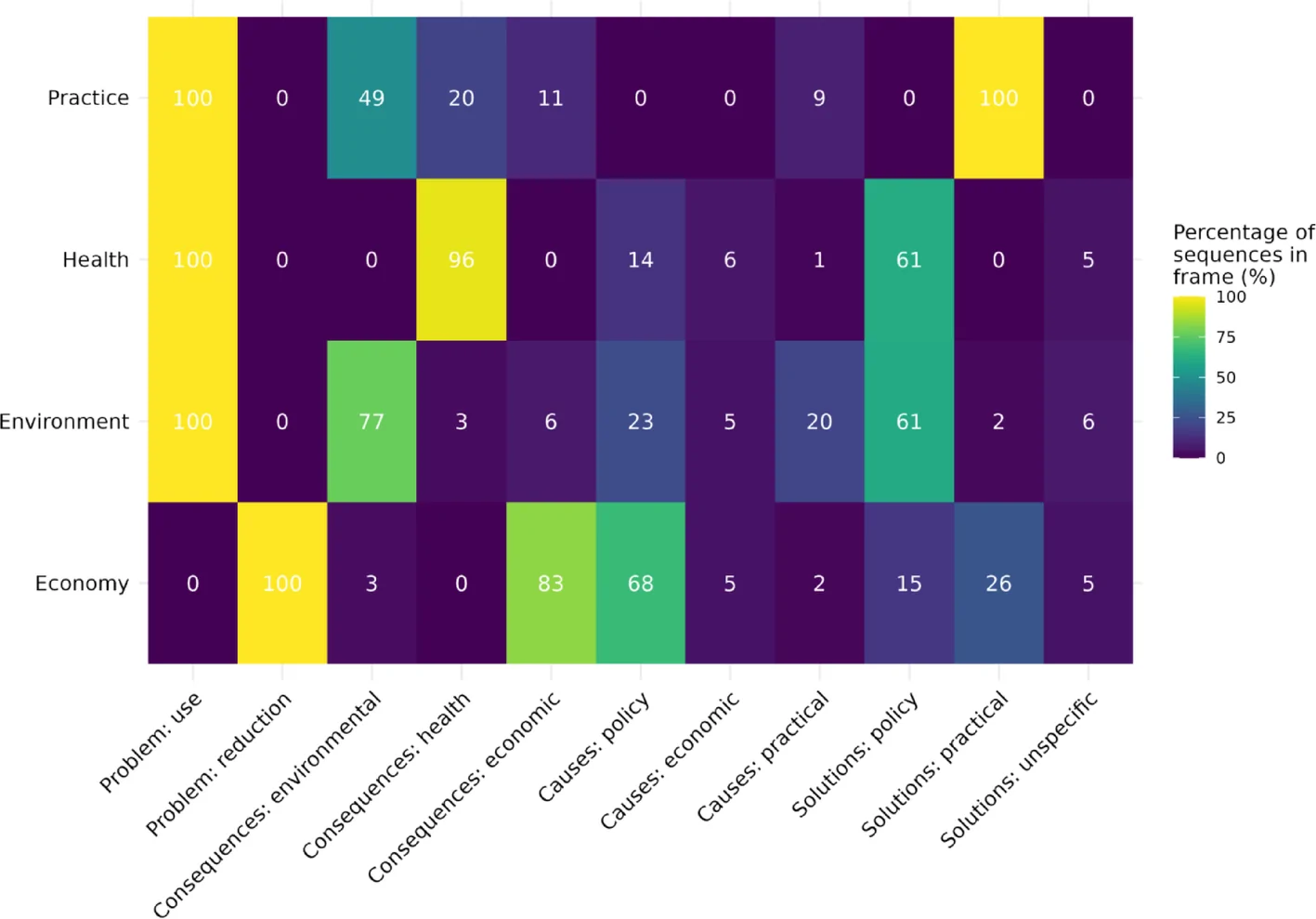
Results of the cluster analysis. The numbers indicate the percentages of frame sequences in a particular cluster (frame) where a specific element was present. The different categories of frame elements are mutually exclusive, meaning that a frame sequence can include, for example, either a practice-related solution or a policy-related solution but not both

Based on the clustering of the 588 frame elements identified in the 293 frame sequences, we identified four frames that were present in public discourse on the issue of pesticide risk reduction in Switzerland: an economy, environment, health, and practice frame. Each frame was characterised by a specific pattern of frame elements (Fig. 2). Three of the four frames (environment, health, and practice) focus on risks of pesticide use, while only the economy frame addresses risks of reducing pesticide use. It is important to emphasise that not all speakers necessarily agree with the frame to which they contribute. However, even when they dismiss the underlying problem, for example by questioning negative health consequences, they still contribute to the health frame through such statements.

The environment frame revolves around negative environmental consequences of pesticide use, such as the contamination of water bodies, harm to nontarget species (e.g. bees and birds), and the accumulation of pesticide residues in soil. Causes were regularly addressed as either policy- or practice-related. Examples of the former included inadequate approval procedures and insufficient application regulations, while examples of the latter included improper or abusive use of pesticides. The mentioned solutions were mostly related to policy, typically regarding stricter rules for the authorisation and evaluation of pesticides. The environment frame was the most salient in our dataset, to which 37% (109) of the frame sequences contributed.

Similarly, the health frame primarily addressed negative consequences of pesticide use, such as poisoning, respiratory issues, neurological disorders, cancer risks, and pesticide residues in food and drinking water. This frame placed little emphasis on causes, instead focusing on policy solutions, which included stricter authorisation procedures, application regulations, and the recognition of pesticide-related illnesses among farmers as occupational diseases. The health frame accounted for 28% (83) of the frame sequences in the dataset.

The practice frame was the last to address the risks of pesticide use. Unlike the environmental and health frames, this frame did not focus on any particular consequences. Although environmental consequences were mentioned most frequently, health and economic consequences were also mentioned occasionally. The primary emphasis, however, was on practical solutions, such as pesticide-reduction measures that farmers could take. Examples included adopting organic farming methods, using pest-resistant crop varieties, and employing technologies such as pest pressure prediction models or advanced machinery for mechanical weed removal. The practice frame was the least salient in our dataset, accounting for only 12% (36) of the frame sequences.

The economy frame was the only frame to address the risks of reducing pesticide use. The frame emphasised economic consequences, such as yield loss, rising food prices, reduced food security, and increasing compliance costs for farmers. In contrast to the other frames, causes were frequently mentioned and were usually related to policy. These included existing or proposed bans on pesticides, stricter approval processes for new active substances, and stricter regulations regarding pesticide use. Examples of the latter included measures such as requirements for designated filling and washing stations. When solutions were mentioned, they tended to be practice-rather than policy-oriented. However, when (proposed) policies were identified as the cause of the problem, their prevention or removal was typically implicitly proposed as the solution. The economy frame accounted for 22% (65) of the frame sequences in the dataset.

### Evidence use in framing

**Fig. 3:**
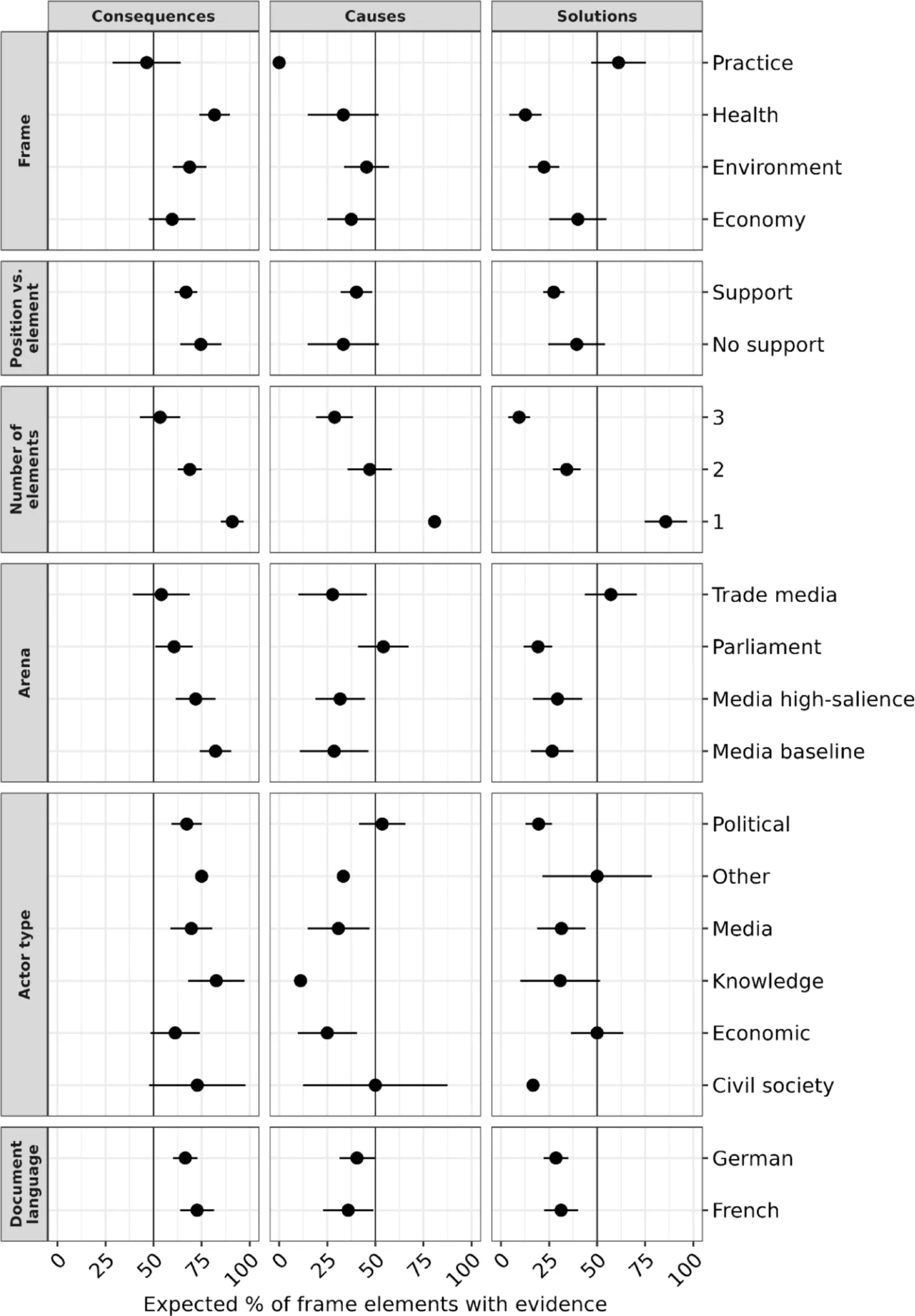
Model results (average marginal effects)

Figure 3 displays the full model results. The main result is reported in the top panel. That is: when examining how evidence was used to frame the pesticide issue, we found that, overall, it was most likely to support consequence elements, less likely to support cause elements, and least likely to support solution elements. However, there were differences between the frames.

For the environment frame, we found a 69% (95% confidence interval: 60%, 77%) likelihood of supporting evidence being presented when consequences were mentioned, but only a 45% (34%, 57%) likelihood when causes were mentioned, and an even lower likelihood of 22% (14%, 30%) when potential solutions were mentioned. This means that in Switzerland, knowledge about environmental consequences was considered most relevant when pesticide use was framed as an environmental issue. Notably, although this aligned with the frame’s emphasis on consequences, evidence for solutions was relatively unlikely to be presented, given how frequently policy solutions were referenced in this frame (see Fig. 2).

For the health frame, we observed a similar pattern (Fig. 3): an 82% (74%, 90%) likelihood of supporting evidence being presented when health consequences were mentioned, a 33% (15%, 52%) likelihood when causes were mentioned, and only a 13% (4%, 21%) likelihood when solutions were mentioned. Given the frame’s strong emphasis on health consequences, this is not surprising. However, although most of the frame sequences that contributed to the health frame also mentioned policy responses, it was unlikely that supporting evidence was provided.

In contrast, for the practice frame, we found only a 46% (29%, 94%) likelihood of evidence being used to support a consequence of pesticide use mentioned in a respective frame sequence. The likelihood was 0% for causes and 61% (47%, 75%) for practical solutions, reflecting the different elements emphasised by the frame, with practical solutions to reduce pesticide risks being by far the most prevalent.

For the economy frame—the only one that emphasised risks of reducing pesticide use—we found a 60% likelihood (CI 48%, 72%) of consequences being supported by evidence, a 38% (CI 25%, 50%) likelihood of causes being supported, and a 40% (CI 25%, 55%) likelihood of solutions being supported. Similar to the health and environment frames, the economy frame strongly emphasised consequences, which corresponded with a similarly high likelihood of consequence-related evidence being used. Unlike the other frames, the economy frame also placed considerable emphasis on policy-related causes of risks associated with reducing pesticide use. However, this emphasis was not reflected in the comparatively low likelihood of evidence being used to support such claims. Compared to the health and environment frames, solutions were given less emphasis in the economy frame. However, when solutions were addressed, it was more likely that evidence was used in their support.

In addition to examining the influence of frames on the use of evidence for different frame elements, we also controlled for the effects of a number of other factors. First, we accounted for the speaker’s position vis-à-vis the frame element mentioned (e.g. whether the speaker supported or opposed the health claim that glyphosate is carcinogenic). Although we found no substantial differences between positions across frame elements, evidence was somewhat likelier to be used for solution elements that were not supported by the speaker (Fig. 3, second panel). Second, we controlled for the number of frame elements mentioned in a frame sequence (Fig. 3, third panel). Given that our data collection focused solely on frame sequences that contained evidence, the probability of evidence use approached 100% when only one frame element was mentioned. However, when two or three elements were present, the likelihood decreased, particularly for solutions and, to a lesser extent, for causes, but not as much for consequences. Consequently, in frame sequences that included consequences, causes, and solutions, evidence use was most likely for the consequence element and least likely for the solution element. Third, we examined the influence of the arena on evidence use for different frame elements (Fig. 3, fourth panel). Similar to the frames, distinct patterns emerged across arenas. In trade media, evidence for solution elements was most likely, which could be attributed to the prevalence of the practice frame in this arena—suggesting that the arena variable primarily reflects compositional differences in framing rather than an independent arena effect. In the parliamentary arena, evidence was equally likely to be used for consequences and causes, but less so for solutions. Compared to the other arenas, the likelihood of evidence use for causes was highest in the parliamentary arena. In the mass media arena, there was little difference between the baseline and the high-salience samples, with evidence use being most likely for consequences in both. However, in the high-salience sample, there was a slight reduction in the likelihood of evidence being used for consequences. Fourth, we controlled for the influence of the speaker’s actor type (Fig. 3, fifth panel). As expected, we observed differences in evidence use across actor types. Notably, knowledge actors, such as scientists and research institutions, were most likely to use consequence-related evidence. In contrast, economic actors, including farmers, had a relatively high likelihood of using solution-related evidence. Finally, we found no substantial differences between documents written in French and German, hinting at no differences in evidence use across languages or regions in Switzerland (Fig. 3, sixth panel).

## Discussion

We set out to understand how evidence was used to frame the issue of pesticide risk reduction in Switzerland in 2013–2022. We based our investigation on the assumption that evidence is employed strategically to support a frame’s key elements, thereby attributing relevance to specific bodies of knowledge in policymaking. The results confirmed that evidence was not likely to be used equally across all frame elements. Confirming our expectation, we found that evidence use differed systematically between frames and was most likely for the element that was most strongly emphasised. For the health, environment, and economy frames, these elements were consequences, whereas for the practice frame, they were solutions. Therefore, it is not surprising that consequences were likely to be supported by evidence. However, causes and policy solutions often lacked such support, even when they were central to a frame. For example, policy solutions were frequently mentioned in sequences contributing to the health and environmental frames, yet it was relatively unlikely that evidence was used in their support. We argue that there are several possible, mutually reinforcing explanations for this observation.

First, solution-oriented knowledge, such as knowledge about policy-related causes and potential policy solutions, was probably less available than problem-oriented knowledge, such as knowledge about the health, environmental, and economic consequences of pesticide use. Research typically concentrates on identifying and quantifying problems, producing a substantial body of knowledge about consequences (Lang & Wiek, 2022). This was reflected in our findings, with knowledge actors (i.e. scientific professionals/experts) being more likely to use evidence to substantiate claims about consequences than claims about causes or solutions. Although understanding problems is crucial, it is equally important to evaluate the suitability and effectiveness of potential responses, including policy measures. However, there has been a noted lack of solution-oriented research (Tengö & Andersson, 2022). Our analysis showed that knowledge about practical solutions to pesticide risk reduction, often used as evidence for the practice frame, did not appear to resonate with more policy-oriented frames, even though most pesticide policies ultimately affect farming practices. This limited transfer of knowledge highlights the need for systematic research on the effectiveness, suitability, and acceptance of policy instruments for pesticide risk reduction. Simultaneously, it is important to recognise that incorporating context-specific and experiential knowledge could help inform decision-making and support effective policy implementation (Fazey et al., 2006).

Second, space and time constraints, particularly in the media arenas but also in parliament, may have limited the amount of information speakers could convey. This potentially prompted them to strategically select which frame elements to support with evidence. In such contexts, the speakers’ primary goal is often to maximise the persuasiveness and credibility of their statements (Cairney, 2016; Majone, 1989). As our results showed, speakers tended to prioritise consequence claims over causes and solutions. Arguably, this was not only because more (and potentially more robust) knowledge about consequences was available but also because consequences are typically more tangible, immediate, and easy to understand. Compared to causes or solutions, which may be complex or, in the case of policy proposals, somewhat speculative, consequences provide a clearer link between problems and their real-world impacts. When supported by evidence, speakers probably see consequences as more effective in capturing attention and enhancing the persuasiveness of their framing. To better understand their reasoning, future research could examine what shapes speakers’ choices to use evidence in support of one frame element over another.

Finally, framing is central to the policymaking process because it allows actors to link a problem and its causes and consequences with a possible solution. However, whether a speaker supports or opposes policy change shapes how the problem is defined (Koon et al., 2016). In our case, proponents of change focused on the risks of pesticide use, while opponents highlighted the risks of reducing pesticide use. Our results showed that supporters of change primarily emphasised the health and environmental risks of pesticide use, whereas opponents stressed the economic risks linked to the reduction of their use. In both cases, speakers focused their framing on the consequences of either maintaining or changing the status quo, drawing attention away from causes or solutions.

In this context, causes were usually mentioned mainly to highlight perceived shortcomings or excesses in current policy or practice, thereby reinforcing the urgency of the problem. For example, proponents tended to attribute pesticide residues in water bodies to inadequate regulation, while opponents generally identified regulations as cost drivers for farmers. However, because both frames ultimately treated consequences as the core of the problem to be addressed, it is not surprising that evidence was most likely used to support consequence claims rather than cause claims. As our findings showed, this held true even when speakers rejected a particular framing (e.g. when the negative health consequences of pesticide use were contested or when positive economic effects of a pesticide ban were highlighted). Moreover, contested consequences can even increase the likelihood of evidence being used to support such claims. The high likelihood that we observed of evidence being used in relation to health consequences supports this, with studies either affirming or rejecting a link between glyphosate and cancer being cited particularly often (Benbrook, 2019).

Despite the close conceptual link between policy-related causes and solutions (see point one), their roles in framing differ. Causes are part of the problem discourse, which focuses on defining the policy issue, while solutions belong to a separate discourse that builds upon the former (Weible & Sabatier, 2018). In terms of the policy cycle, problem discourse corresponds to the agenda-setting stage, while solution discourse relates to the policy formulation stage (Howlett & Ramesh, 1995). In this context, the two popular initiatives—one proposing a general ban on synthetic pesticides and the other restricting subsidies to pesticide-free farms—narrowed the public debate and focused attention on specific solutions. As we have shown elsewhere (Reber et al., 2025b), this shift led to the increased use of solution-oriented evidence by opponents of change, reflected in a greater likelihood of evidence being used for solutions within the economy frame and when a solution element was opposed. In contrast, proponents of change continued to focus on the problem discourse, as reflected in the greater likelihood of evidence being used for consequences within the health and environment frames. Although the negative consequences of pesticide use were potentially considered to be more tangible, and therefore more effective for mobilising support (see point two), it is also possible that proponents lacked credible evidence demonstrating the effectiveness and benefits of the policy solutions and popular initiatives (Bystricky et al., 2020; Schmidt et al., 2019).

## Conclusion

Our findings reinforce the argument that evidence is used strategically to support specific elements of a frame, making framing a key factor in determining which bodies of knowledge are relevant to policymaking. Although other factors also influence how speakers use evidence in political discourse (Hofmann et al., 2023), our analysis showed that the likelihood of evidence use varied across frame elements depending on the frame. Specifically, we found that across all frames, the most emphasised element was also the one most likely to be supported by evidence. However, consequence claims were generally likelier to be backed by evidence than claims about causes or policy solutions. This pattern probably reflects several factors: the limited and imbalanced availability of problem- and solution-oriented knowledge, space and time constraints that compelled speakers to focus on what they considered the most persuasive elements, and the problem-oriented nature of political discourse, which revolves around competing definitions of what the issue actually is about.

Examining which elements of a frame are likely to be supported by evidence allowed us to draw conclusions about the types of knowledge actively mobilised by speakers in political discourse in Switzerland. For example, in the case of pesticide risk reduction, evidence is likely to consist of knowledge about potential health risks or practical solutions to reduce those risks. Conversely, a framing perspective on evidence use can help identify the kinds of knowledge that are absent or underutilised in relation to a policy issue. In our case, this included the limited use of evidence for policy solutions to address environmental and health concerns regarding pesticide use. However, the absence of such evidence in public discourse does not necessarily mean that the knowledge is unimportant or irrelevant for policymaking, but it may be unavailable, inaccessible, or overlooked.

Recognising the role of framing in shaping evidence use can support a more transparent and reflexive approach to policymaking that acknowledges the interplay between evidence and framing as an integral part of the policy process (Schweber et al., 2015). This could provide a basis for reflecting on both the knowledge required and the knowledge actually used to define and address an issue (Greenhalgh & Russell, 2009; Head, 2010). For researchers, policymakers and the media as gatekeepers, this would mean paying close attention to how evidence is used to frame an issue, and by whom (Hawkins & Ettelt, 2019). It also means ensuring that institutions at the science–policy interface facilitate the integration, cumulation, and evaluation of diverse bodies of knowledge (Hofmann et al., 2025), while taking into account the frames they resonate with as interpretive lenses (Moerschel et al., 2023). As we have shown, adopting a frame-sensitive approach to evidence-informed policymaking helps to unveil gaps in the knowledge used to define problems and develop solutions. This, in turn, enables both researchers and policymakers to recognise and address uncertainty, ultimately leading to better-informed decisions.

## Data Availability

The data and R code for replicating the analysis are available at https://doi.org/10.5281/zenodo.15687871.

## Appendix

### Number of documents/frame sequences

See Table 1.

**Table 1:** Overview of document corpora and samples: total number of documents mentioning pesticides per arena (corpus), number of documents sampled and manually checked for evidence use, number of checked documents containing at least one frame sequence with evidence, and total number of frame sequences with evidence (maximum of three per document) recorded in the manual content analysis of frame sequences (Step 2)

	Number of documents (n)			Number of frame sequences with evidence (n)
	Corpus	Sampled	Sampled with evidence	
Mass media				
Baseline	2973	230	59	79
High-salience	301	141	50	70
Total	3274	371	109	149
Trade media	5642	239	41	47
Parliament	1686	234	84	97
Total	10,602	844	234	293

### Number of frame elements by category

See Table 2.

**Table 2:** Number of frame elements per category

	Number of frame elements (n)			
	Total	Consequences	Causes	Solutions
Evidence use				
Evidence used	288	180	50	58
No evidence used	300	83	78	139
Frame				
Practice	68	28	4	36
Health	158	82	21	55
Environment	227	96	55	76
Economy	135	57	48	30
Position				
Supporting problem/solution	479	208	107	164
Not supporting problem/solution	109	55	21	33
Number of frame elements				
1	69	45	8	16
2	306	147	49	110
3	213	71	71	71
Arena				
Mass media baseline	139	73	21	45
Mass media high-salience	139	64	41	34
Trade media	90	37	18	35
Parliament	220	89	48	83
Actor type				
Civil society	23	11	6	6
Economic	115	49	28	38
Knowledge	45	23	9	13
Media	117	56	26	35
Other	19	8	3	8
Political	269	116	56	97
Document language				
French	187	84	39	64
German	401	179	89	133
Total	588	263	128	197

### Regression coefficients

See Table 3.

**Table 3:** Model results (log odds, standard errors in parentheses). Reading example for ‘frame: environment’, the coefficient indicates the effect of the frame being ‘environment’ rather than ‘economic’ (reference category) on the log odds of using evidence

	Evidence use
Frame element: causes	3.82** (1.64)
Frame element: consequences	1.86* (1.05)
Frame element: solutions	3.96* (2.20)
Frame: environment	− 0.16 (0.57)
Frame: health	− 0.47 (0.70)
Frame: practice	− 14.56 (673.15)
Position: support	− 0.06 (0.61)
Number of frame elements	− 1.30*** (0.41)
Arena: media high-salience	− 0.05 (0.73)
Arena: parliament	1.20 (1.05)
Arena: trade media	0.34 (0.91)
Actor type: economic	− 1.75 (1.08)
Actor type: knowledge	− 2.80* (1.54)
Actor type: media	− 1.35 (1.09)
Actor type: other	− 1.84 (1.76)
Actor type: political	− 1.01 (1.25)
Document language: German	− 0.05 (0.49)
Frame element: consequences × frame: environment	0.76 (0.71)
Frame element: solutions × frame: environment	− 0.57 (0.85)
Frame element: consequences × frame: health	1.54* (0.84)
Frame element: solutions × frame: health	− 1.22 (1.01)
Frame element: consequences × frame: practice	13.93 (673.15)
Frame element: solutions × frame: practice	14.87 (673.15)
Frame element: consequences × position: support	0.14 (0.73)
Frame element: solutions × position: support	− 1.28 (0.80)
Frame element: consequences × number of frame elements	0.60 (0.49)
Frame element: solutions × number of frame elements	− 0.71 (0.60)
Frame element: consequences × arena: media high-salience	− 0.28 (0.88)
Frame element: solutions × arena: media high-salience	0.40 (0.97)
Frame element: consequences × arena: parliament	− 2.82** (1.29)
Frame element: solutions × arena: parliament	− 0.22 (1.37)
Frame element: consequences × arena: trade media	− 1.11 (1.05)
Frame element: solutions × arena: trade media	0.67 (1.09)
Frame element: consequences × actor type: economic	2.24 (1.37)
Frame element: solutions × actor type: economic	2.83 (2.17)
Frame element: consequences × actor type: knowledge	4.14** (1.82)
Frame element: solutions × actor type: knowledge	3.32 (2.51)
Frame element: consequences × actor type: media	1.84 (1.36)
Frame element: solutions × actor type: media	1.98 (2.18)
Frame element: consequences × actor type: other	2.63 (2.11)
Frame element: solutions × actor type: other	2.64 (2.67)
Frame element: consequences × actor type: political	2.33 (1.59)
Frame element: solutions × actor type: political	0.93 (2.34)
Frame element: consequences × document language: German	− 0.17 (0.59)
Frame element: solutions × document language: German	0.29 (0.67)
Log likelihood	− 297.68
McFadden’s pseudo R2	0.27
Area under the curve (AUC)	0.83

**p* < .1; ***p* < .05; ****p* < .01

## Footnotes

1 Note that we did not assess the accuracy of the evidence identified, as a systematic fact-check would have been both impractical and unrepresentative of how evidence is typically treated in real-world political discourse, which seldom involves such verification. In other words, our focus was on analyzing how evidence was used, rather than evaluating the evidence itself (Yanovitzky & Weber, 2020).

2 Using regular expressions, we searched for the following terms: *pesti(c|z)id, inse(c|k|t)ti(c|z)id, (h)?erbi(c|z)id, f(u|o)ngi(c|z)id*, plant protection product, and *pflanzenschutzmittel, produit(s)? phytosanitaire, prodott(i|o) fitosanitari.*

3 Blick (German), Le Temp (French), Neue Zürcher Zeitung (German), Tages-Anzeiger (German), and Tribune de Genève (French).

4 BauernZeitung (German), Die Grüne (German), and Agri (French).

5 The first author and two trained coders did the coding of the documents. Since we considered only frame sequences that included evidence, the coders were first asked to identify evidence sequences (i.e. frame elements supported by evidence). Once this had been done, they were asked to identify, for each sequence, the speaker, the problem, and any additional frame elements mentioned by the same speaker. Based on the coding of 26 mass media, trade media, and parliamentary documents, when each coder’s coding was compared with the author’s coding, the average level of agreement was satisfactory at 77% for the identification of evidence sequences and 100% for the identification of speakers in the identified sequences (i.e. 77% agreement for the identification of frame sequences). The average agreement (Krippendorff’s alpha (α)) was satisfactory for all content analysis variables: α = 1 for problem type, α = 0.76 for consequence element, α = 0.87 for cause element, α = 0.84 for solution element, α = 0.72 for speaker’s position regarding the frame element, α = 0.69 for evidence goal (i.e. the frame element the evidence referred to), and α = 0.80 for actor type. Although α ≥ 0.67 is generally deemed acceptable (Krippendorff, 2018), the evidence coding was reviewed by the author and corrected as necessary. To further improve data quality, the coders met weekly to discuss and agree on ambiguous cases, aiming to ensure consistent and reliable coding.

6 As mentioned already, we defined evidence use as the use of factual knowledge by a speaker to ‘prove’ that a particular claim is true or valid (Majone, 1989; Yanovitzky & Weber, 2020). For the purpose of this analysis, a vague assertion like ‘science shows that pesticides are dangerous’ or referring to personal experience like ‘I didn’t experience any yield loss since I stopped using pesticides’ did qualify as evidence use.

7 We also recorded the valence of the evidence used (i.e. whether it supported, contradicted, or was ambivalent regarding the speaker’s position on a frame element; Yanovitzky & Weber, 2020). However, we found very few instances of contradictory or ambivalent evidence and, therefore, did not include them in the model (Step 4). This indicated that evidence in the Swiss pesticide discourse is primarily used strategically to support particular positions, rather than as a subject of debate. The evidence itself is rarely contested.
